# Box–Behnken Design: Wet Process Optimization for Saponins Removal From *Chenopodium quinoa* Seeds and the Study of Its Effect on Nutritional Properties

**DOI:** 10.3389/fnut.2022.906592

**Published:** 2022-07-01

**Authors:** Khadija El Hazzam, Manal Mhada, Mohamed Louay Metougui, Kamal El Kacimi, Mansour Sobeh, Moha Taourirte, Abdelaziz Yasri

**Affiliations:** ^1^Biodiversity and Plant Sciences Program (BPS), AgroBioSciences Department (AgBS), Mohammed VI Polytechnic University (UM6P), Benguerir, Morocco; ^2^Laboratory of Research in Sustainable Development and Health, Chemical Sciences Department, Faculty of Science and Technology, Cadi Ayad University (UCA), Marrakech, Morocco; ^3^Agricultural Innovation and Technology Transfer Center (AITTC), AgroBioSciences Department (AgBS), Mohammed VI Polytechnic University (UM6P), Benguerir, Morocco; ^4^Industrial Executive Operations Division, Gantour Industrial Site, Act 4 Community Gantour, OCP, Youssoufia, Morocco

**Keywords:** quinoa seeds, wet process, saponins elimination, optimization, box-behnken design, nutritional quality

## Abstract

The pseudocereal grain, Quinoa (*Chenopodium quinoa* Willd.), has a great nutritional value due to its high contents of proteins, fiber, minerals, and vitamins. However, saponins naturally present outside the grains represent an obstacle to their consumption as human food. Before consumption, the grains are subjected to various treatments, which alter their nutritional value. In an attempt to eliminate the maximum of saponins using the wet process, while minimizing the washing conditions and preserving the nutritional quality, we explored the effects of several parameters, including volume of water, treatment time, soaking time, number of washing, and water temperature, followed by an optimization process using Box–Behnken Design, and finally, the impact of this process on the physicochemical and techno-functional properties of six quinoa genotypes seeds was evaluated. As a result, the variation of the treatment time, volume, and temperature of the water positively affected the saponins leaching. According to the quadratic model, the maximum percentages of eliminated saponins (96.53%−96.77%) were found at a temperature of 50°C, treatment times from 60 to 69 min, and water volumes from 6.99 to 7.50 mL per gram of seeds. The optimized method did not affect the proteins and microelements content (Zn, Mn, B, Mo), while a slight decrease of macro-elements (K, P, Ca, S, Mg) was noted in the level of some genotypes. On the other hand, a significant improvement of the techno-functional properties such as water and oil holding capacity was noted, with a sharp drop-in emulsifying activity in all genotypes without affecting the standard values of pH (6.4–6.8) and moisture content (10%−11%) of the seeds. Hence, the optimized method showed to be a more potential method for saponins removal than the currently used dry method.

## Introduction

Quinoa (*Chenopodium quinoa* Wild.) is a pseudocereal native to the Andean region and cultivated for about 7000 years (1). The plant is considered one of the most nutritious food crops whose grains provide a protein value similar to milk casein (2), with close protein digestibility corrected amino acid score (PDCAAS) values varying between 0.85 and 0.89 for raw quinoa and from 1.00 to 1.09 for washed quinoa (3). Furthermore, the seeds' protein content ranges from 12 to 23% and includes all essential amino acids (1). Quinoa contains fewer carbohydrates than most common cereals (wheat, corn, barley, rice, rye, and sorghum), considered a good alternative for diabetics (4), and starch is the most important carbohydrate (5). Quinoa is also an excellent source of dietary fiber that ranges between 7.0 and 9.7% (5). Moreover, quinoa can also be considered an oilseed due to the quality and volume of its lipid fraction, which can reach up to 9.5% (4). The mineral fraction in raw quinoa seeds ranges from 2.0 to 3.4% and is rich in macro-elements, such as P, K, Ca, and S, and microelements, such as Fe, Zn, Mo, B, and Mn (5, 6). These constitutional elements give quinoa excellent functional properties such as solubility, water retention capacity, gelling, emulsification, and foam formation, allowing for diverse uses (1).

However, saponins, the most abundant secondary metabolites with about 86% at the outer layer of the seeds, remain an obstacle since they constitute an antinutritional factor and give a bitter taste to the grain. In addition, all saponins are potentially toxic at high concentrations due to their hemolytic activity, which requires their elimination before the consumption of seeds or their processing to manufacture food products (7).

Several research works have focused on saponins removal techniques. In short, wet, dry, and genetic methods have been evaluated over the past 20 years (7). In addition, dry methods such as extrusion, roasting, and mechanical abrasion were also thoroughly studied, especially their optimization, effect on the elimination of saponins, and the nutritional quality of the seed after treatment. Several studies have suggested a medium level of saponin removal with an improvement in some properties. Despite that, at certain levels, the application of these methods destroys the shape of the seed and affects the nutrient quantities, especially minerals (8–10). Oppositely, few studies have addressed the effect of wet processes on changes in the chemical composition of the seed.

This work aims to evaluate the parameters influencing the leaching of saponins from quinoa seeds to give a general view of the wet process. Furthermore, the selected parameters were used to optimize the elimination of saponins with a minimum of washing conditions using an experimental design. Finally, the impact of the optimized process on the nutritional quality of the seeds is assessed by studying their physicochemical and techno-functional properties.

## Materials and Methods

### Plant Materials

Six different quinoa genotypes (Ames 13727, Q2, Ames 13761, Ames 22157, NSL 106398, and Titicaca) were cultivated at the experimental farm of Mohamed VI Polytechnic University in Benguerir, Morocco, whose geographical coordinates are 32 ° 14 ′ north, 7 ° 57 ′ west, and the altitude equal to 449 meters. The seeds of the studied quinoa genotypes, both washed and not, were ground using a Waring 8010ES blender for 1 min. The samples were then subjected to different analyzes, reported in detail in the respective sections.

### Wet Process Method

The genotype Ames 13727 was selected for the initial screening step due to its high content of saponins compared to other genotypes. Five parameters were used for the washing process screening. These include the number of washing times (1–5), the volume of water (3, 5, 7, 9, 11 ml/g of seeds), treatment time as a time of processing under stirring (15, 30, 60, 120, 150 min), soaking time as a time of moistening to aid in seed coat removal without stirring (15, 30, 60, 120, 150 min), and water temperature (20, 30, 40, 50, 60°C).

The experimental data for the screening step were obtained by leaching saponins from the quinoa seeds under different conditions. In each case, four parameters were kept in a fixed value (Temperature at 40°C, treatment time under stirring at 30 min (350 rpm), the volume of water equal to 7 mL/g of seeds, with a single wash), with the variation of the fifth parameter according to its variables.

The wet process was carried out for the optimization step by following the conditions mentioned in Table 1, with a single wash and without a soaking step. Then, the seeds were dried for 24 h at 40°C in a drying oven (Memmert Universal Oven U, Germany) and then grounded. Finally, the quinoa powder was stored at 4°C until its use.

**Table 1:** Box–Behnken design for the experiments of %ES.

**Std**	**Run**	**Factors**			**%ES**		
		**X_**1**_ (**°**C)**	**X_**2**_ (min)**	**X_**3**_ (mL/g)**	**Actual value**	**Predicted value**	**Residual**
1	15	50(-1)	30(-1)	7(0)	93.36	93.36	−0.0055
2	1	60(+1)	30(-1)	7(0)	93.33	93.36	−0.0320
3	9	50(-1)	90(+1)	7(0)	95.68	95.65	0.0320
4	12	60(+1)	90(+1)	7(0)	96.97	96.96	0.0055
5	10	50(-1)	60(0)	5(-1)	93.38	93.67	−0.2942
6	11	60(+1)	60(0)	5(-1)	94.18	94.45	−0.2677
7	14	50(-1)	60(0)	9(+1)	95.42	95.15	0.2678
8	13	60(+1)	60(0)	9(+1)	95.99	95.69	0.2943
9	5	55(0)	30(-1)	5(-1)	89.88	89.58	0.2998
10	8	55(0)	90(+1)	5(-1)	92.03	91.77	0.2623
11	3	55(0)	30(-1)	9(+1)	89.93	90.19	−0.2622
12	6	55(0)	90(+1)	9(+1)	93.59	93.89	−0.2997
13	4	55(0)	60(0)	7(0)	95.35	95.49	−0.1460
14	2	55(0)	60(0)	7(0)	95.64	95.49	0.1460
15	7	55(0)	60(0)	7(0)	95.49	95.49	0.0000

*Experimental and predicted values for the quadratic model*.

*X_1_, Water Temperature (°C); X_2_, Treatment time (min); X_3_, Volume of water (mL)*.

*%ES: percentage of eliminated saponins*.

### Physico-Chemical and Techno-Functional Characterization of Washed (WS) and Non-washed Quinoa Seeds (NWS)

#### Mineral Profile

The analysis of the mineral profile was realized according to the method of Mhada et al. (6). First, multi-elemental trace analysis was performed using Agilent 5110 ICP-OES (GBO15A). Both macro-elements (K, P, S, Mg, Ca) and microelements (Fe, Zn, Mn, B, and Mo) were analyzed. The quantification was done using single element ICP standards TraceCERT^®^ with a 1 g/L concentration.

#### Scanning Electron Microscopy Observations

The observations of different quinoa seed compartments and layers were carried out using an electron microscope. First, a scalpel was used to prepare median longitudinal sections of seeds, and then the samples were mounted and fixed on metal stubs using commercial adhesive. Next, the seeds were carbonized for 5 min using an automatic carbon cord coater. To determine the relative abundances and spatial distributions of K, Ca, Mg, P, and Fe, the samples of WS and NWS were analyzed using scanning electron microscopy (SEM, supra 55vp-Zeiss, Germany) coupled to an Energy Dispersive X-ray (EDX) analyzer. These analyses were conducted at CAC facilities (Center of Analysis and Characterization, Marrakech, Morocco). The SEM-EDX conditions were a temperature of −10°C, pressure of −50 Pa, and voltage of 20 kV.

#### Protein Content

The Kjeldahl method for organic nitrogen was used to measure the protein content according to the official analysis methods of AOAC International using a Kjeltec 2300 autoanalyzer (11). The total nitrogen content was determined, and the protein content was quantified using the quinoa's specific conversion factor equal to 5.75 (12). The measurements of each treatment were made in triplicates.

#### Saponins Content

The saponins extraction method was based on Nickel et al. (8), with modifications reported by Mhada et al. (6). The analysis of saponins was carried out spectrophotometrically according to the method applied by Irigoyen and Giner (13). The OD reading was done at 528 nm by a Spectrophotometer (Thermo Scientific™ 840-300300). The quantification was achieved with the Quillaia saponin assay (Y0001564 Merck) (*r*^2^ = 0.9914). The results were expressed in kg of saponins per 100 kg of dry seeds. All measurements were made in triplicates. The percentage of eliminated saponins (%ES) compared to raw seeds was calculated using the following equation (1):


(1) $$\begin{array}{r} \%ES=100-(A\times 100)/B \end{array}$$

Where A represents the saponins content in WS and B represents the saponins content in NWS.

#### Total Phenolic Content (TPC)

The TPC was determined using the Folin–Ciocalteu method (14). Summarily, 1 mL of Folin–Ciocalteu reagent (HC97724201 Merck) was added to 200 μL of the sample. After 5 min of incubation, 800 mL of Na_2_CO_3_ were added, the mixture was vortexed, incubated for 30 min, then the absorbance was measured at 750 nm. The TPC was expressed in milligrams of gallic acid equivalents per gram of quinoa flour (mg GAE/g).

#### Physicochemical and Functional Properties

##### Water Holding Capacity (WHC)

WHC was evaluated using the method of Pellegrini et al. (15). In brief, 10 mL of water was added to 1 g of sample. Then, the mixture was vortexed for 1 min. After the centrifugation of the samples (6,000 rpm, 15 min), the supernatant was weighed. The WHC was expressed in g of water contained per g of quinoa flour.

##### Oil Holding Capacity (OHC)

OHC was determined using Pellegrini et al. (15) method with slight modifications. In short, 1 g of flour from each treatment was suspended with 10 ml of corn oil (density ¼ 0.92 g/ml) and vortexed for 1 min, then centrifugated for 15 min at 6,000 rpm. Finally, the OHC was calculated using the following equation (2):


(2) $$\begin{array}{r} OHC\;(\text{g}/\text{g})\;=\;(P0-P1)/m0 \end{array}$$

Where m_0_ represents 1 g of the sample, P_0_ represents the weight of 10 ml of corn oil, and P_1_ represents the weight of free corn oil.

##### Swelling Capacity (SWC)

SWC was presented as ml of increase of volume per g of quinoa flour. The mixture of 1 g of quinoa flour and 10 mL of distilled water was heated to 95°C for 30 min. After cooling to room temperature, centrifugation was carried out at 6,000 rpm for 15 min. Finally, the supernatant was removed and the pellet was weighed (16).

##### Emulsifying Activity (EA)

To analyze the EA (15), 1 g of quinoa flour was homogenized with 50 ml of distilled water for 30 s at 6,000 rpm using laboratory agitators (IKA^®^ EuroStar 20 Digital, Germany), then 50 ml of corn oil was added and homogenized a second time for 1 min. After centrifugation (6,000 rpm for 5 min), the emulsion volume was measured and the EA was calculated using the following equation (3):


(3) $$\begin{array}{l} \text{EA}\text{\% }=\;(\text{volume}\;\text{of}\;\text{emulsified}\;\text{layer}\;/\text{volume}\;\text{of}\;\\ \;\text{whole}\;\text{suspension}){\;}^{*}100 \end{array}$$

##### pH and Moisture Content

The pH was measured according to Pellegrini et al. (15) and the moisture was measured using the method 925.10 (17).

#### Statistical Analysis and Optimization Procedure

SPSS Software (22.0) was used to perform statistical analysis. The results were expressed as the Mean ± Standard Deviation (SD) of three parallel trials. Comparison of means was realized by one-way analyzes of variance (ANOVA). Moreover, the Principal Component Analysis (PCA) and Pearson correlation test were conducted using R software (v 4.1.0.). The data were considered statistically significant if the *p*-values were <0.05 and <0.01 for the correlation test.

The response surface methodology (RSM) was used to optimize the wet process of saponins elimination from quinoa seeds. The statistical study was performed by Design-Expert 12 software (Stat-Ease, Minneapolis, MN, USA). It was used for regression and graphical analysis of experimental data of %ES. A Box–Behnken Design (BBD) was used to optimize the number of experiments required to investigate this study which was 15. Wherefore, the experimental sequence was randomized to minimize the effects of the uncontrolled factors. Indeed, the design center point was repeated three times to estimate errors and curvature. After processing, the %ES from the seeds was opted out as the response-dependent term.

## Results and Discussion

### Screening of Wet Process Parameters

**Figure 1:**
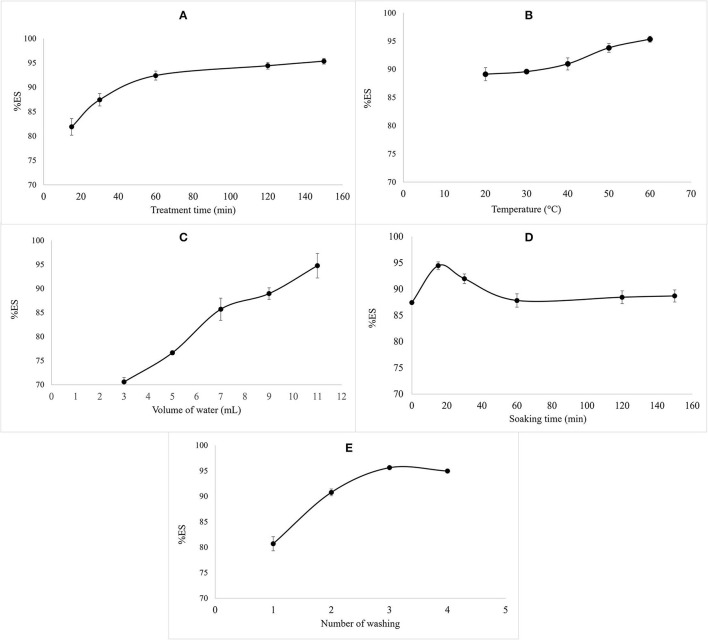
The effect of variation of treatment time **(A)**, water temperature **(B)**, volume of water **(C)**, soaking time **(D)**, and number of washing times **(E)** on %ES.

According to the results illustrated in Figure 1, the variation of the volume and the temperature of water used during the treatment and the treatment time positively affected the percentage of saponin leaching. At the beginning of the process, the elimination of saponin from the seeds is rapid. Then, after 1 h of stirring, the total saponin concentration in the quinoa seeds reached an asymptotic value, as shown in Figure 1A. Similarly, Quispe et al. (18) reported very rapid saponins leaching at the beginning of the process with an asymptotic value of saponin content in the quinoa seeds, and Vega-Galvez et al. (19) estimated that the minimum washing time required to extract most of the saponins is 60 min.

Concerning the effect of water temperature, a positive correlation between the temperature and %ES was noted in Figure 1B. The same tendency was noted with the volume of water (Figure 1C). This positive correlation is justified by saponins' surfactant properties, which allow their solubilization in water and, consequently, their elimination (20). Temperatures above 60°C and treatment times of more than 90 min caused an alteration of the structure of the seeds.

Regarding the soaking time before washing, it had a negative effect on the elimination of saponins after 15 min of soaking. This negative effect is due to the increase of saponin content detected in the seeds (Figure 1D). The same results were reported by Nickel et al. (8), suggesting the irrelevance of soaking during the process due to the effect of this parameter on the release of saponins which are inside the seed during extraction, which means their increase during quantification.

In the same line, the application of several washes on the seeds does not affect saponin elimination. Thus, 80% of the saponins were removed from the first wash. In addition, alteration of the seed morphology was observed after the third washing (Figure 1E).

### Optimization of the Wet Procedure Using Response Surface Methodology

#### Statistical Analysis and Model Fitting

The experimental points for the BBD were carried out based on the results of the section screening of wet parameters. Water temperature (X_1_, °C), treatment time (X_2_, min), and volume of water (X_3_, (mL/g)) were chosen as the independent variables. The low, middle, and high levels of each variable (in coded forms −1, 0, and +1, respectively) are present in Table 1.

The quadratic model was chosen as the BBD model to determine the regression equation that predicts the %ES. From a first overview of the BBD matrix, we noticed that the maximum, average, and minimum responses were 96.97, 94, and 89.88%, respectively, and their max/min ratio was 1.09.

**Figure 2:**
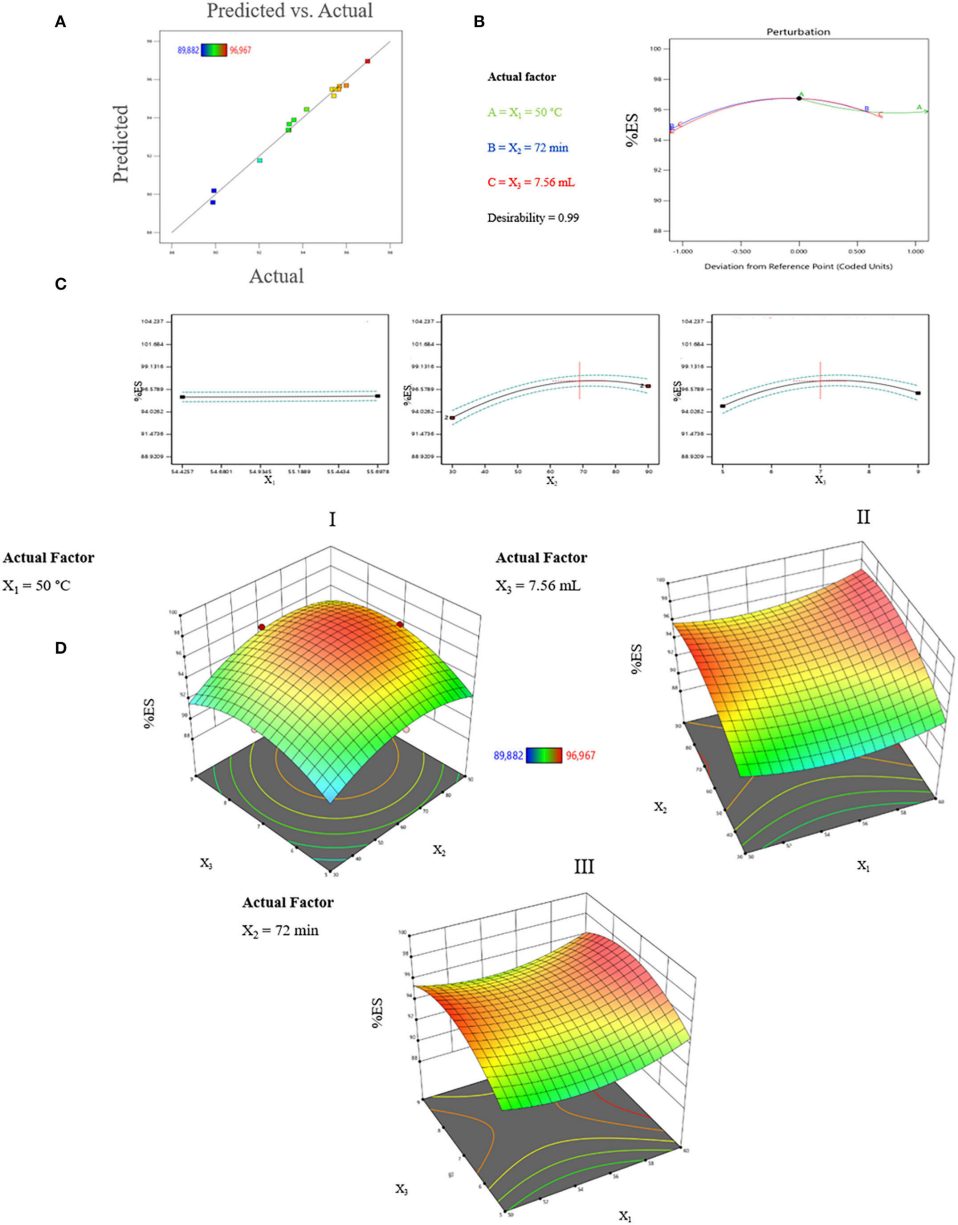
**(A)** Graphical representation of the observed values as a function of predicted values (%). **(B)** Perturbation of optimal conditions. **(C)** Forecast profile of optimal conditions for %ES. X_1_ = water temperature; X_2_ = treatment time; and X_3_ = quantity of water. **(D)** Response surface plots of the interaction effect between parameters on %ES during processing; DI, The effect of the interaction between treatment time and volume of water; DII, The effect of the interaction between water temperature and treatment time; DIII, the effect of the interaction between water temperature and volume of water.

To evaluate the effect of the three factors, we have analyzed our data matrix by multiple linear regressions. The actual and predicted values are represented in Table 1 and Figure 2A. According to the correlation graph, the actual and predicted values are close, and all residual values are <2σ. Figure 2A demonstrates a good correlation between the values observed and those predicted with a determination coefficient *R*^2^ equal to 0.9893, indicating an important correlation between responses and independent variables with only 1.07% of the total variable not fitted by the quadratic model. The Predicted R^2^ of 0.8374 agrees with the Adjusted *R*^2^ of 0.9699, with a difference of <0.14 (Table 2). The adequate precision measured the signal-to-noise ratio as equal to 24.533, it is >4, indicating an adequate signal where this quadratic model can be used to navigate through the design space.

**Table 2:** ANOVA for response surface quadratic model of %ES.

**Source**	**SS**	**DF**	**MS**	***F* value**	***p*-value**	**Sinification**
**Model**	62.50	9	6.94	51.17	0.0002^c^	Significant
**X**_**1**_	0.8672	1	0.8672	6.39	0.0527	Not significant
**X**_**2**_	17.32	1		127.60	<0.0001^c^	Significant
**X**_**3**_	3.74	1	3.74	27.54	0.0033^b^	Significant
**X**_**1**_ **X**_**2**_	0.4277	1	0.4277	3.15	0.1360	Not significant
**X**_**1**_ **X**_**3**_	0.0139	1	0.0139	0.1026	0.7617	Not significant
**X**_**2**_ **X**_**3**_	0.5730	1	0.5730	4.22	0.0950	Not significant
**X**_**1**_^2^	6.85	1	6.85	50.49	0.0009^c^	Significant
**X**_**2**_^2^	15.07	1	15.07	111.05	0.0001^c^	Significant
**X**_**3**_^2^	16.49	1	16.49	121.51	0.0001^c^	Significant
**Residual**	0.6785	5	0.1357			
**Lack of Fit**	0.6359	3	0.2120	9.94	0.0927	Not significant
**Pure Error**	0.0426	2	0.0213			
**Cor Total**	63.18	14				
**Adeq Precision**	24.5333					
**C.V.%**	0.3918					
**Std. dev**.	0.3684					
**Mean**	94.01					
***R*** ^2^	0.9893					
**Adjusted** ***R***^2^	0.9699					
**Predicted** ***R***^2^	0.8374					

*X_1_, Water Temperature (°C); X_2_, Treatment time (min); X_3_, Volume of water (mL)*.

*SS, Sum of squares; MS, Mean of Square; C.V, Coefficient variation*.

*^a^significant at 0.05 level*;

b *significant at 0.01 level*;

c *significant at 0.001 level*.

The ANOVA was utilized to verify the significance and relevance of the model. The model's F-value of 51.17 implies that it is significant, with only a 0.02% chance that this large F-value could occur due to noise (Table 2). Moreover, the *p*-values of X_2_, X_3_, ${\text{X}}_{1}^{2}$, X${}_{2}^{2}$, and ${\text{X}}_{3}^{2}$, lesser than 0.05, indicate significant model terms. In addition, the mean square of lack-of-fit equal to 9.94, with *p*-value of 0.0927 corresponding to an insignificance value, supports the model's validity.

#### Equation of the Model

The equation from the fitted quadratic model representing the %ES in the function of the coded variables is as follows (4):


$$\begin{array}{l} \%\text{ES}=95.49+0.3292X_{1}+1.47X_{2}+0.6835X_{3}+0.3270X_{1}X_{2}\;\\ \;-0.0590X_{1}X_{3}+0.3785X_{2}X_{3}+1.36X_{1}^{2}\;\\ \;-2.02X_{2}^{2}-2.11X_{3}^{2} \end{array}$$

where X_1_ = water temperature; X_2_ = treatment time; X_3_ = volume of water. The equation of the established model shows that all the factors have a positive correlation with the %ES from quinoa seeds.

#### Prediction Profiler

The profiler makes the possibility to show the impact of factors on responses. This analysis considers the interaction between the factors to find the optimal conditions for a response using desirability functions. According to Figure 2C, X_2_ and X_3_ showed a positive effect on %ES between 30 min and 72 min for X_2_ and between 5 and 7.56 ml/g for X_3_. Moreover, X_1_ showed no effect between 50 and 60°C. The %ES increased with processing time and the volume of water to reach its maximum under the following conditions: X_1_= 50 °C, X_2_ = 72 min, and X_3_ = 7.56 ml/g. The profile shows that the desirability is close to 1 and %ES equals 96.74% (Figure 2B). This elimination rate is greater than that found by Vega-Galvez et al. (19), who reported an elimination of 96% after 2 h of processing, and the one found by Irigoyen and Giner (13), who reported an %ES of 80%.

#### Effect of Process Parameters Interactions on %ES

The exploitation of the graphically validated model was carried out by plotting the Response Surfaces 3D of interaction impacts on %ES. Figure 2DI represents the interaction between the volume of water and treatment time when the temperature of water was fixed at 50°C. Figure 2DII illustrates the interaction between water temperature and treatment time with 7.56 mL as a fixed water volume. Finally, the interaction between temperature and volume of water during 72 min as a fixed treatment time is represented in Figure 2DIII.

The variation of water volume used during the treatment and the treatment time are the most influential factors with a positive correlation in the interval of 5 to 7.50 mL for X_3_ and 30 to 70 min for X_2_. Figure 2DI shows that setting the temperature at 50°C and varying the treatment time from 55 to 80 min and water volume between 6.60 and 8 mL for each gram of seeds makes it possible to obtain the maximum of %ES which reached a value of 96.77% under X_1_ = 50°C, X_2_ = 68.86 min, and X_3_ = 7.43mL. Figures 2DII and 2DIII show that the leaching of saponins increases with the increase of the treatment time and the volume of water until a maximum is obtained at 69 min for X_2_ and 7.46 mL for X_3_. These results are confirmed in the forecast profiler and model equation. At the same time, the temperature did not show a noticeable effect in the range of 50–60°C.

#### Optimization of the Variables

The optimal conditions for the washing process were provided by the digital optimization tool of the design software. Nine different solutions were chosen considering the minimum amount of water to use, respecting ecological and commercial interests, and having a maximum of %ES. Numerical optimization results are given in Table 3. The studied factors were within their ranges, justifying the chosen interval for each experimental parameter. By fixing the temperature at 50°C and varying the treatment time between 60 and 69 min and the volume of water between 6.99 and 7.50 mL per gram of seeds, we can obtain the maximum of %ES (96.53–96.77%).

**Table 3:** Numerical optimization of quadratic model.

**Number**	**Temperature**	**Treatment time**	**Quantity of water**	**% ES**	**Desirability**
**Goal**	In range	In range	In range	maximize	maximize
**1**	50	60.216	6.991	96.53	0.966
**2**	50	68.857	7.43	96.773	0.986
**3**	50	64.405	7.239	96.715	0.982
**Goal**	minimize	In range	In range	maximize	maximize
**1**	50	68.028	7.466	96.768	0.991
**2**	50	66.413	7.411	96.757	0.99
**3**	50	65.977	7.329	96.75	0.99
**4**	50	67.247	7.175	96.74	0.989
**Goal**	minimize	minimize	minimize	maximize	maximize
**1**	50	46.301	5.996	94.762	0.795
**2**	50	45.583	6.03	94.739	0.795

#### Validation of the Model

To validate the model, experimental tests were performed using the predicted parameters. A washing test was performed in triplicate using 6.99 ml of water heated to a temperature of 50°C for each gram of quinoa seed for 60.22 min. The predicted optimum value of %ES was 96.53%, with the best model desirability equal to 0.97. This result agrees with that obtained experimentally (96.69%) (Table 4). Therefore, Box–Behnken Design and the obtained model were accurate and decisive tools for describing and modeling the wet process of removing saponins.

**Table 4:** Experimental model validation.

**Parameters**	**X_**1**_**	**X_**2**_**	**X_**3**_**				
**Optimum conditions**	50	60.22	6.99				
**Response**	**Predicted Mean**	**Predicted Median**	**Observed**	**Std Dev**	**95% PI low**	**Data Mean**	**95% PI high**
**%ES**	96.53	96.53	96.69	0.37	95.63	96.69	97.43

*X_1_, Water Temperature (°C); X_2_, Treatment time (min); X_3_, Volume of water (mL)*.

### Characterization of Washed Quinoa Seeds

#### Effect of Optimized Process on Mineral Profile

**Figure 3:**
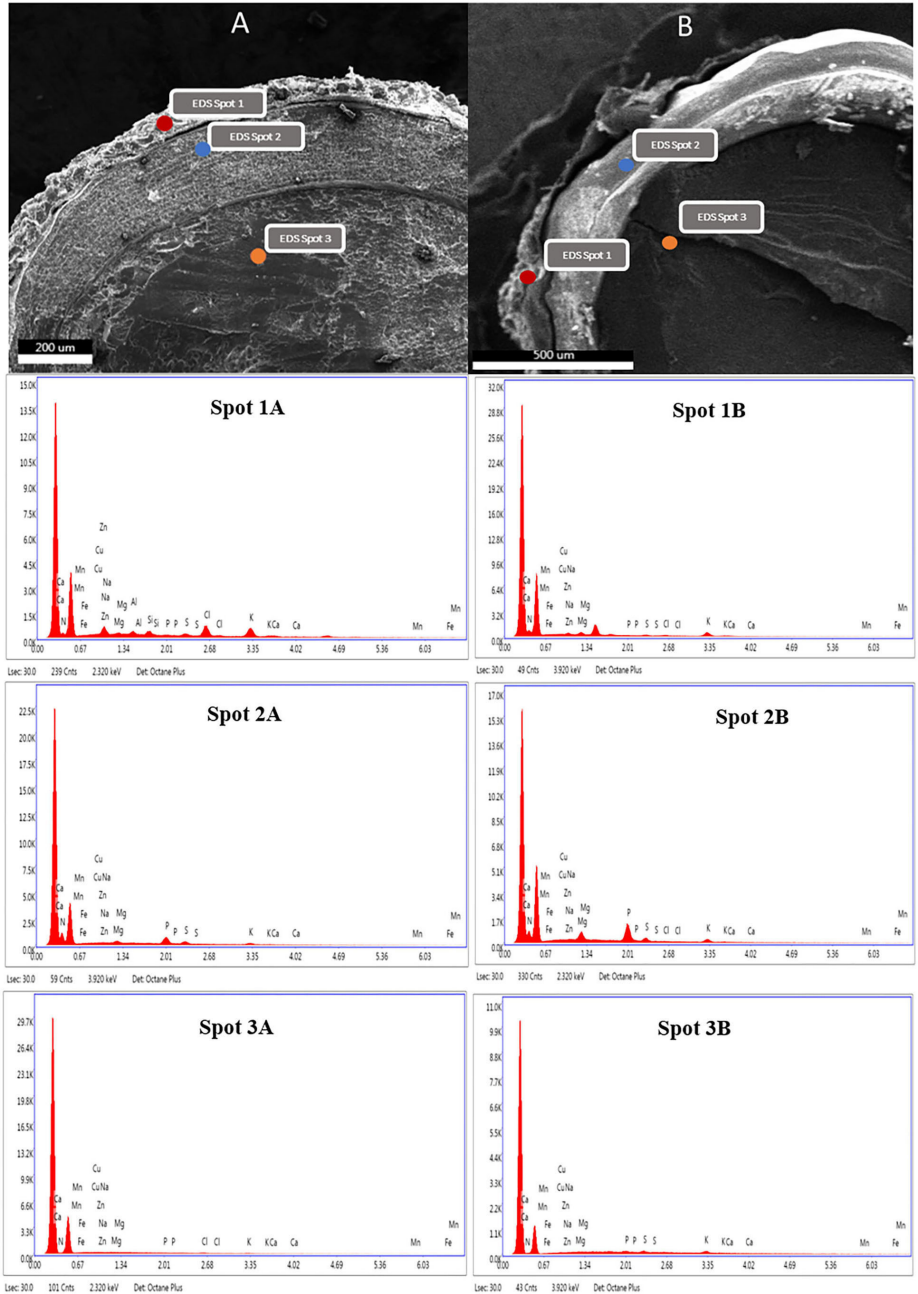
SEM photographs of C. quinoa and EDX distribution of macro and microelements in the layers of non-washed **(A)** and washed **(B)** quinoa seeds. (Spot 1: pericarp, Spot 2: cotyledon, Spot 3: perisperm).

The effect of the optimized wet process on macro and microelements of six genotypes of quinoa seeds are shown in Table 5 and Figure 3. The processing induced a negative effect on the macro-elements content. The most affected element was potassium. The loss magnitude varied widely between quinoa genotypes, from a 28% reduction for Titicaca to 70% for Ames 13727. The loss of potassium can be explained by its high water solubility and its position in the seeds (21, 22). Figure 3 shows that potassium is mainly concentrated at the pericarp level (**Spot 1A**) against the cotyledon (**Spot 2A**) and the perisperm (**Spot 3A**). After washing, a significant loss was noted at the level of the pericarp (**Spot 1B**). For phosphorus, seed washing induced a significant decrease in three genotypes, reaching up to 40, 19, and 16% in Titicaca, Q2, and NSL 106398 genotypes, respectively, with a non-significant effect in the remaining genotypes. Figure 3 shows that the phosphorus is localized at the cotyledon level. The elimination of phosphorus can indicate phytates removal during processing, which means that the loss of this element is due to the action of water on phytates, a form of hydro-soluble phosphorus storage in quinoa seeds (22). Likewise, a significant decrease in calcium, sulfur, and magnesium was recorded after washing. EDX analysis shows that Ca, S, and Mg are found more in the pericarp of the seeds (**Spot 1A**) than the interior (**Spot 2A, 3A**), which facilitates their loss during the process. According to Konishi et al. (22), K, Mg, Fe, and Zn are generally present in the form of phytate at the level of quinoa which explains the strong correlation of these elements with phosphorus (**Figure 5**). Regarding the microelements, the optimized process did not affect the Zn, B, and Mo contents. In contrast, a significant decrease in the Mn content was observed for most genotypes, and an increase in the Fe content was noted in all genotypes. Wang et al. (10) reported a decrease of 7% of Ca, 12% of Fe, 15% of Zn, and 3% of Mg after the peeling treatment of quinoa seeds. Mhada et al. (6) studied the polishing effect of Titicaca variety seeds on macro and microelements, the results showed a loss of 43.52% of K, and 22.63% of Mg compared to 28% of K, and 18.75% of Mg in our case. For Zn, Mn, and B, the polishing effect was significant with a decrease of 7.5%, 39% and 39.16% successively, while the washing effect was non-significant. The levels of the mineral elements Mo and S in the Titicaca variety were shown to be unaffected by both methods. Both polishing and washing of quinoa grains reduced minerals to a certain extent compared to wholemeal flour (23), but the loss level was greater using polishing than washing.

**Table 5:** Variation of mineral concentrations, protein content, saponin content, and TPC in raw and processed quinoa seeds genotypes.

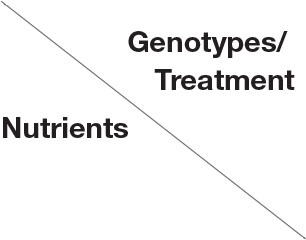			**Ames 13727**		**Q2**		**Ames 13761**		**Ames 22157**		**NSL 106398**		**Titicaca**	
		**NWS**	**WS**	**NWS**	**WS**	**NWS**	**WS**	**NWS**	**WS**	**NWS**	**WS**	**NWS**	**WS**
Macro-elements (g/kg)	K	23.00 ± 0.70^a^	6.90 ± 0.70^b^	15.50 ± 0.90^a^	6.80 ± 0.40^b^	17.00 ± 1.20^a^	6.6 ± 0.60^b^	16.9 ± 1.20^a^	6.70 ± 0.50^b^	16.70 ± 0.70^a^	0.64 ± 0.10^b^	7.10 ± 0.50^a^	5.10 ± 0.30^b^
	P	5.70 ± 0.90^a^	4.90 ± 0.50^a^	5.30 ± 0.40^a^	4.30 ± 0.30^b^	5.10 ± 0.40^a^	4.70 ± 0.50^a^	4.90 ± 0.40^a^	4.40 ± 0.30^a^	5.10 ± 0.40^a^	4.30 ± 0.10^b^	3.80 ± 0.20^a^	2.30 ± 0.20^b^
	S	2.30 ± 0.30^a^	1.80 ± 0.10^b^	2.00 ± 0.20^a^	1.60 ± 0.00^b^	2.10 ± 0.20^a^	1.70 ± 0.10^b^	2.10 ± 0.20^a^	1.60 ± 0.10^b^	2.00 ± 0.10^a^	1.50 ± 0.00^b^	1.20 ± 0.10^a^	1.30 ± 0.10^a^
	Mg	3.50 ± 0.90^a^	2.20 ± 0.30^b^	2.80 ± 0.30^a^	1.90 ± 0.10^b^	3.00 ± 0.40^a^	2.10 ± 0.20^b^	3.00 ± 0.40^a^	2.00 ± 0.20^b^	2.90 ± 0.30^a^	1.80 ± 0.00^b^	1.60 ± 0.10^a^	1.30 ± 0.10^b^
	Ca	2.60 ± 0.30^a^	1.00 ± 0.10^b^	1.20 ± 0.10^a^	0.70 ± 0.00^b^	2.50 ± 0.30^a^	1.10 ± 0.10^b^	2.30 ± 0.20^a^	0.70 ± 0.10^b^	1.80 ± 0.20^a^	0.60 ± 0.00^b^	1.43 ± 0.10^a^	1.10 ± 0.10^b^
Micro-elements (g/kg)	Fe	137.50 ± 6.36^a^	59.67 ± 7.23^b^	129.33 ± 9.24^a^	49.00 ± 6.00^b^	135.33 ± 1.15^a^	58.67 ± 8.14^b^	133.67 ± 9.61^a^	55.00 ± 4.58^b^	114.33 ± 2.89^a^	55.00 ± 5.57^b^	59.00 ± 1.41^a^	62.00 ± 1.41^b^
	Zn	36.50 ± 2.12^a^	36.33 ± 3.79^a^	33.67 ± 4.16^a^	35.00 ± 2.00^a^	35.67 ± 3.21^a^	36.00 ± 2.00^a^	35.00 ± 2.65^a^	33.33 ± 1.53^a^	32.00 ± 2.00^a^	33.00 ± 1.00^a^	22.67 ± 3.06^a^	27.67 ± 1.53^a^
	Mn	26.33 ± 2.08^a^	12.67 ± 1.53^b^	19.00 ± 1.41^a^	14.00 ± 1.00^b^	27.50 ± 2.12^a^	13.00 ± 1.00^b^	25.50 ± 0.71^a^	11.33 ± 0.58^b^	28.50 ± 2.12^a^	12.33 ± 0.58^b^	21.00 ± 1.41^a^	20.00 ± 1.73^a^
	B	35.27 ± 1.84^a^	30.75 ± 2.46 ^b^	32.64 ± 1.53^a^	29.01 ± 0.18^b^	32.85 ± 1.63^a^	29.82 ± 1.77^a^	33.93 ± 1.80^a^	31.50 ± 2.59^a^	34.18 ± 1.47^a^	32.00 ± 2.64^a^	29.43 ± 1.00^a^	31.46 ± 2.78^a^
	Mo	0.31 ± 0.11^a^	0.32 ± 0.01^a^	0.31 ± 0.05^a^	0.26 ± 0.00^a^	0.27 ± 0.00^a^	0.25 ± 0.03^a^	0.38 ± 0.01^a^	0.35 ± 0.02^a^	0.35 ± 0.04^a^	0.26 ± 0.02^b^	0.18 ± 0.02^a^	0.22 ± 0.04^a^
Proteins (g/100 g)		18.46 ± 0.08^a^	16.06 ± 2.83^a^	18.15 ± 2.79^a^	15.96 ± 1.93^a^	14.82 ± 0.32^a^	15.04 ± 0.61^a^	14.49 ± 0.63^a^	15.58 ± 1.81^a^	14.38 ± 1.95^a^	14.40 ± 0.37^a^	11.58 ± 1.08^a^	13.34 ± 2.37^a^
Saponins (g/100 g)		8.15 ± 0.75^a^	0.27 ± 0.01^b^	2.23 ± 0.01^a^	0.12 ± 0.05^b^	3.81 ± 0.02^a^	0.13 ± 0.03^b^	6.74 ± 0.04^a^	0.18 ± 0.01^b^	6.42 ± 0.11^a^	0.24 ± 0.01^b^	2.03 ± 0.28^a^	0.19 ± 0.02^b^
TPC (mg GA eq. /100 g)		147.66 ± 5.35^b^	188.09 ± 11.12^a^	141.92 ± 0.38^b^	171.66 ± 0.81^a^	147.1 ± 2.65^a^	139.26 ± 2.64^b^	142.19 ± 11.53^a^	144.63 ± 1.97^a^	164 ± 0.7^a^	161.61 ± 14.13^a^	114.20 ± 12.48^b^	162.25 ± 3.46^a^

*Data are expressed as mean ±SD (based on triplicate analyses)*.

a−b *Different superscript letters in the same line for each genotype indicate the significant difference (p < 0.05)*.

*NWS, Non-washed seeds; WS, Washed seeds; TPC, Total phenolic compounds*.

#### Proteins, Saponins, and Polyphenols Contents

The results of the optimized method's effect on the elimination of saponins and preservation of proteins and phenolic compounds are shown in Table 5.

The protein contents vary between 11.58 g/100 g noted in the genotype Titicaca and 18.46 g/100 g noted in the genotype Ames 13727 g/100 g before treatment. Craine and Murphy (3) reported that protein contents range between 10.04 and 13.68 g/100 g of quinoa seeds, and De Bock et al. (24) reported values between 12.50 and 18.80 g/100 g (24). After processing, the values vary from 13.34 g/100 g to 16.06 g/100 g in Titicaca and Ames 13727, respectively, and statistically, the wet process showed no significant effect on the protein content. In accordance with our findings, Ruales and Nair (25) did not reveal any significant change in the quality of the protein content and amino acid composition in quinoa seeds after washing, which affirms that the wet process does not affect the protein nutritional quality of seeds. On the other hand, an increase in protein content was noted in some studies of quinoa seeds processing, especially those that used the dry method (6, 26). Using those methods, we hypothesize that the increase in the protein content is probably due to the loss of the pericarp part, which increases their concentrations during the analysis. The same remark was noted at the level of Titicaca where the content of protein was increased from 11.58 g/100 g in NWS to 13.34 g/100 g in WS. Tumpaung et al. (27) reported that most of the proteins accumulate in the embryo; therefore, the saponin removal process would not affect the nutrient not located at the pericarp (27).

For the effect of the optimized process on saponins content, the six genotypes showed a high level of saponins, which varied between 8.15% in the genotype Ames 13727 and 2.03% in the genotype Titicaca, with a significant difference between genotypes. These percentages of saponins are superior to a safe level for human consumption, hence the importance of treatment before their consumption (8). After processing, a high significant %ES was recorded for all genotypes, varying from 93% for genotype Q2 to 97% for Ames 22157, with saponins content ranging between 0.12% and 0.27%. The elimination of saponins has been highly correlated with their initial seed concentration. Furthermore, the comparison between the wet method used in this study and the polishing method from other studies showed a high effect of the wet process on saponins removal with 96% on average, while the dry process only removed 73% (6, 10).

Regarding the effect on TPC, water washing induced a significant increase in most genotypes, with values varying from 139.26 to 188.09 mg GA /100 g. These results agree with those found by Nickel et al. (8). They reported that the application of the wet processing on quinoa seeds resulted in a significant increase in the TPC after washing under running water. This increase was justified by the release of soluble phenolic compounds under the action of water and temperature. The traditional dry process removes up to 40% of phenolic compounds (10). Similarly, the roasting process removes more than 40% of the phenolic compounds (8). Moreover, according to Caravaca et al. (28), the application of pearling (20% and 30%) as a dry saponin removal process can cause a reduction in the content of free and bound polyphenols by 21.5 and 35.2%, respectively.

#### The Effect of the Optimized Process on Techno-Functional and Physicochemical Properties of Quinoa Seeds

**Figure 4:**
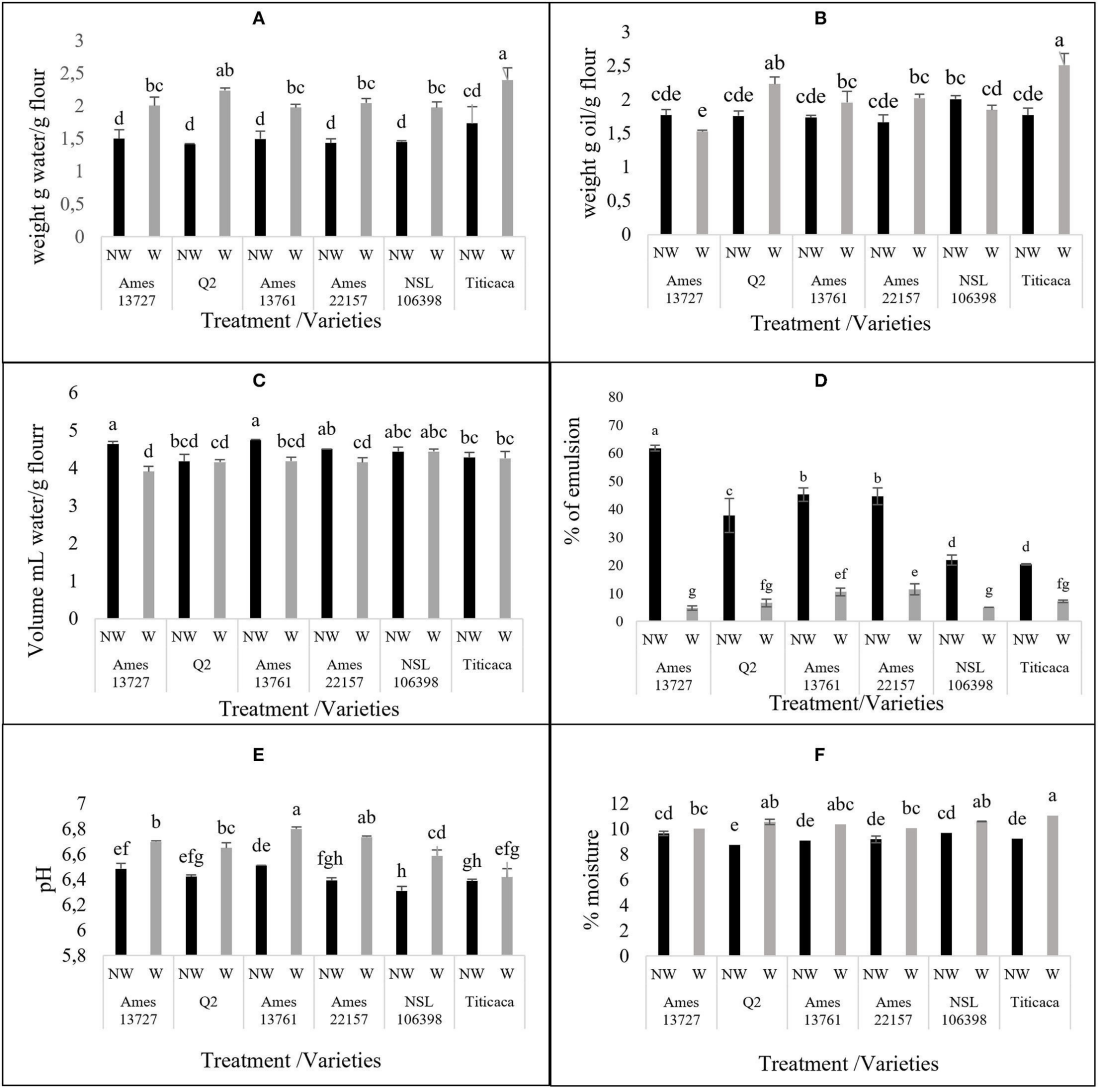
The effect of wet method in techno-functional **(A)** water holding capacity, **(B)** oil holding capacity, **(C)** Swelling capacity, **(D)** Emulsifying activity and physico-chemical **(E)** pH, **(F)** Moisture % properties of different quinoa seed genotypes. NW, non-washed seeds; W, washed seeds.

**Figure 5:**
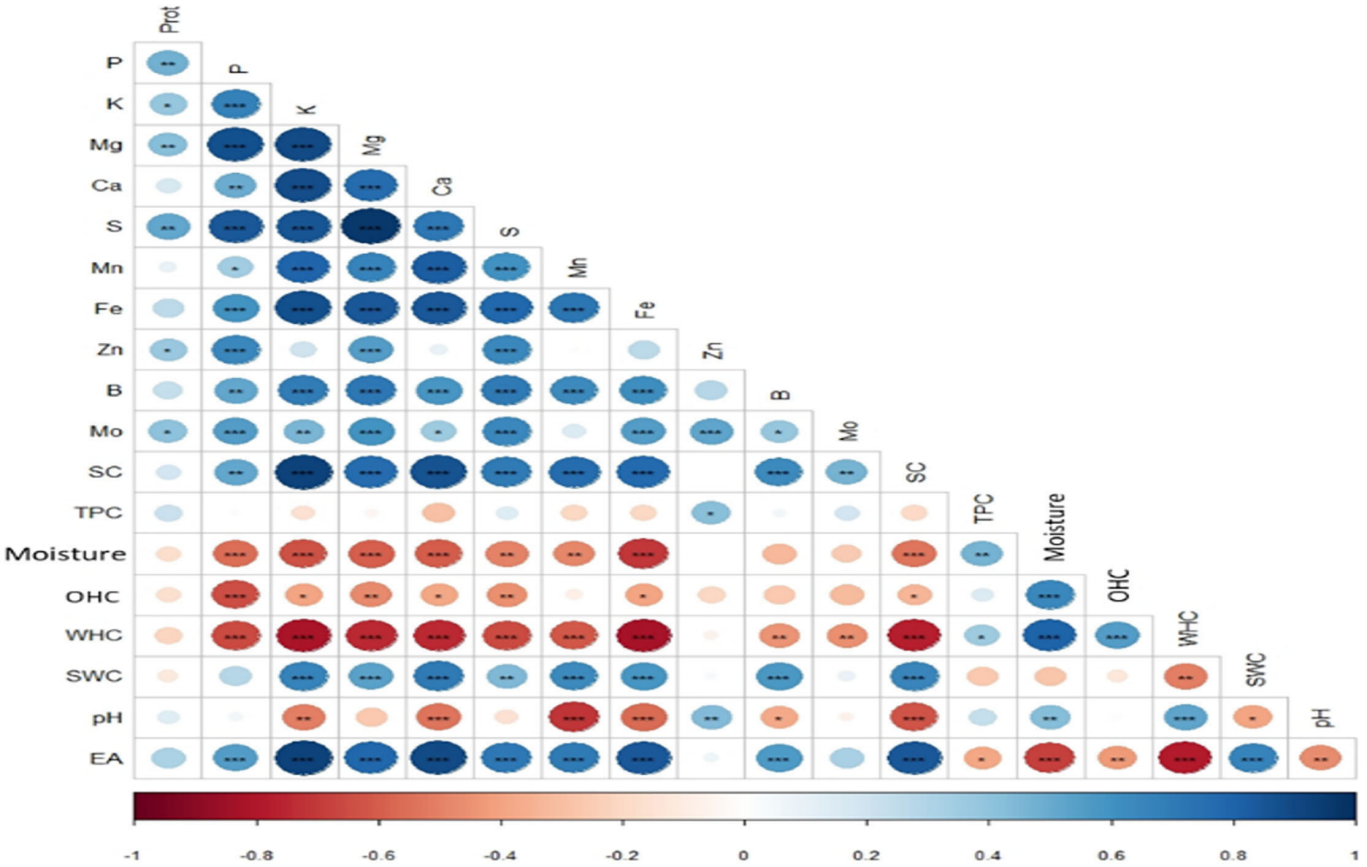
Pearson correlation between different properties during the wet process.

To examine the effect of the optimized process on the nutritional quality of quinoa seeds, the techno-functional (WHC, OHC, SWC, and EA) and physicochemical (pH and moisture content) properties of the seeds have been determined. The results are illustrated in Figure 4. Before treatment, the values of WHC fluctuated between 1.41 and 1.98 g of water/g of flour, with a non-significant difference between genotypes. These values are in agreement with those reported by Ahmed et al. (29) in the order of 1.92 g of water retained per g of sample. Similarly, Pellegrini et al. (15) recorded values of order 1.44 to 1.8 g/g regardless of the genetic material studied. The treatment of quinoa seeds significantly influenced the WHC (Figure 4A). The values of WHC increased after treatment in all genotypes from 32% in Ames 13761 to 58% in Q2 or from 1.50 to 1.98 g/g for Ames 13,761 and 1.41 to 2.24 g/g in Q2. The obtained results showed the same trend compared to those of Wang et al. (10), who reported an increase in WHC ranging from 34 to 37% after a dry process. Similarly, our values agree with the results obtained by Solaesa et al. (30), which reported an increase in the hydration capacity value to 2.29 g/g of flour of the genotype Titicaca after the elimination of saponins with the dry method. The variation in WHC can be explained by several physical and chemical factors, including the application of treatments with high temperatures. For example, cooking and wet roasting increase WHC because of the protein's deployment and consequently the exposure of peptide bonds or other polar side chains, retaining more water molecules (31, 32). However, no significant correlation was found between WHC and protein content (*R* = −0.2147) (Figure 5). On the other hand, starch and lipid content may affect the ability of flour to retain water; a positive correlation has been recorded in the literature between WHC and starch gelatinization under thermal conditions (10). Conversely, the richness of the seeds in lipids can prevent the absorption of water (29, 31).

For OHC property, our results showed a non-significant difference after treatment in most studied genotypes except for Titicaca and Q2, with an increase of 42 and 27%, respectively (Figure 4B). The increase of OHC improves the flavor retention capacity, a highly desirable quality in the food industry (33). However, Aguilera et al. (31) reported that aqueous treatments and temperature did not affect OHC. The variation in OHC is more dependent on the chemical composition of the seeds, including the presence of residues of hydrophobic amino acids, and these non-polar side chains are fat absorption sites (33).

According to Figure 4C, the treatment significantly decreased SWC of the hydrotreated flour compared to the native for the genotypes Ames 3727, Ames 1361, and Ames 22157, whereas in Q2, NSL 106398, and Titicaca genotypes, non-significant differences were observed. Wang et al. (10) suggested a positive correlation of SWC with amylose content, and it is noted that starch in quinoa represents about 58.1 to 64.2% of the dry matter, with 8 to 19% of amylose (34, 35). This could explain the decrease of SWC by the loss of amylose during treatment, which is characterized by high solubility in water (19). Other researchers have suggested that temperature can influence the ability of flour to swell. Perez-Rea and Antezana-Gomez (16) reported that the increase in SWC of quinoa starch starts from 55°C. Also, according to Ruales et al. (23), pre-cooking at 60°C for 20 min leads to starch gelatinization and, consequently, significant increases in SWC values. Moreover, Nayouf et al. (36) found that at room temperature, water readily enters the amorphous regions of the seed and interacts with starch molecules through hydrogen bonds, leading to a slight swelling of the granules; however, this swelling remains reversible, which explains the non-significant difference in the genotypes Q2, NSL 106398, and Titicaca.

Concerning the EA of quinoa, it varied between 61.76 and 20.30% before treatment (Figure 4D). These values are similar to those reported by Delgado and Albarracín (37) for quinoa (52.86%), wheat (49.76%), and soybean (55.90%), and by Aguilera et al. (31), who reported activity of 22.9% for chickpeas and 47.4% for lentils. Washing significantly decreased the EA in all genotypes. The decrease percentage ranged from 65% for Titicaca to 92% for Ames 13727 to attain values varying between 4.76% in Ames 13727 and 10.52% in Ames 13761 after applying the optimized wet process. These values are lower than the one reported by Aguilera et al. (31), who found that the application of hydrotreatment as soaking in water or cooking can negatively affect EA by up to 70%.

On the other hand, Solaesa et al. (30) reported a high EA of 62.5% in the Titicaca genotype after an abrasive polishing of seeds against activity of 7.14% in our case. This suggests the sensitivity of this activity to treatment with water more than to dry treatments. According to Figure 5, a strong positive correlation (*R* = 0.84) was recorded between EA and saponins content, and a low positive correlation (*R* = 0.32) was noted with proteins. This result can affirm that saponins are the origin of this activity, and the high correlation can be explained by the saponins structure, a basic biosurfactant known for its emulsifying role by excellence (38). These results mean the effectiveness of the wet method used to eliminate saponins without affecting the protein content.

For the effect of washing on moisture content and pH (Figures 4E,F), the wet process increased these two properties, which varied between 6.4 and 6.8 for pH and between 10 and 11% for the moisture content, less than the maximum recommended humidity of 13%. Pellegrini et al. (15) reported that pH values varied from 6.42 to 6.63 in white quinoa. Likewise, Miranda et al. (39) reported that the pH values were between 6.18 and 6.40 in quinoa seeds. Therefore, both moisture content and pH values remain within the standards defined by the quinoa Codex Alimentarius proposed by FAO (40).

#### Principal Component Analysis (PCA)

**Figure 6:**
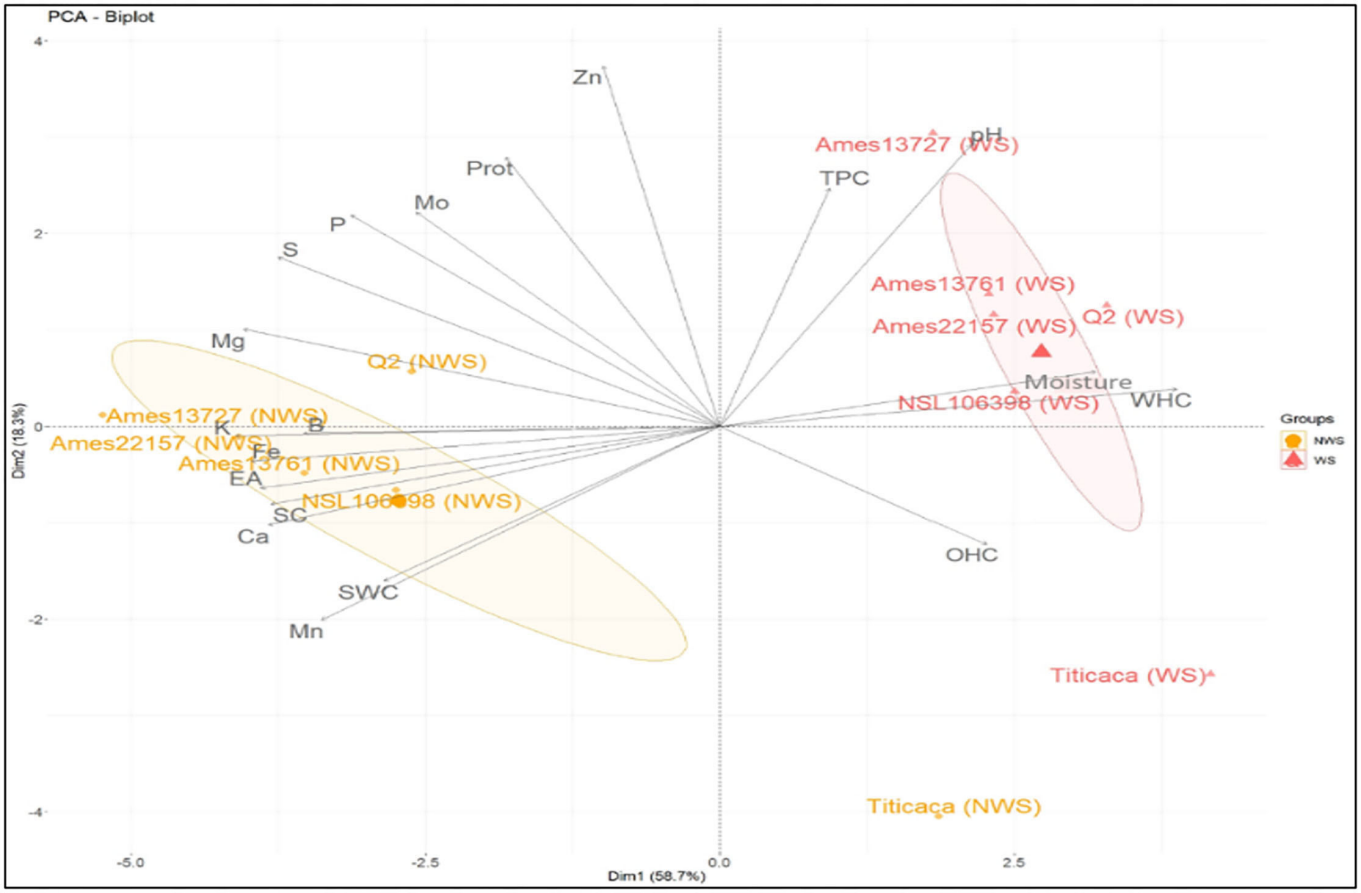
PCA analysis of different genotypes.

PCA was used to show variation among treated and untreated genotypes and identify correlations between the properties (Figure 6). The results were projected into planar space formed by the two first principal components (Dim1 and Dim2). These two dimensions described 58.7% and 18.3%, respectively, of the total variation. The graph shows the similarity between the treated genotypes (Ames 13727, Q2, Ames 13761, Ames 22157, NSL 106398) located closely in the upper-right part of the PCA graph. High values characterize these genotypes in TPC, pH, moisture, and WHC, which explains the positive correlation between these properties at the level of the correlation study shown in Figure 5. However, the untreated genotypes are in the lower-left middle part; these genotypes are richer in K, Fe, B, Mg, S, Ca, and P, in addition to the higher values in saponins with strong EA. As already mentioned, a strong positive correlation was found between these elements due to their solubility in water, their position at the seed level, and the elimination of phytates during the wet process. In contrast, the elements Zn, Mo, and protein content are shown to be less related to genotypes and processing. However, the treated and untreated Titicaca genotype was positioned closer in the right lower part. This genotype was very distinct from other genotypes, with low values for all properties except OHC where the highest value was noted after washing. This genotype appears to be less affected by treatment, which generally explains the seed's morphology. Also, this indicates the necessity of optimizing processing for each group of genotypes based on their genetic pool or seed morphology for optimal results.

## Conclusion

This study examines the effect of five parameters on the wet process for saponins lixiviation throughout an optimization process using the response surface methodology (RSM). Then, the optimized process's effect on the nutritional quality of quinoa seeds of six genotypes was evaluated. The variations in the volume of water, water temperature, and treatment time caused the high leaching of saponin. Minimizing washing conditions to 6.99 ml of water heated to a temperature of 50°C for each gram of quinoa seed for 60.22 min were chosen as optimum conditions to eliminate the maximum of saponins. The optimum method showed a less negative effect on macro-elements than the dry method. For most of the microelements, their contents were conserved after applying an optimized process. The examination of the effect of the wet process on the nutritional quality of quinoa seeds shows an improvement in WHC, TPC, pH, and EA. While, a non-significant effect was noted for proteins, SWC, and OHC in most of the used genotypes. The variation in genetic material showed no difference in the effect of the wet method on physicochemical and techno-functional properties, except for the elimination of saponins, which has been highly correlated with their initial seed concentration. These results suggest that a soft polishing for genotypes with more than 4% of saponin or genotypes with a hard-outer layer, as in the case of Titicaca, will be a good step before the application of an optimized wet process to obtain sweet seeds. The results show the efficiency of the optimized wet process in eliminating saponins with enhancement in specific nutritional properties, thus minimizing the volume of water and energy of the process. Further research can be carried on to combinate this method with the industrial dry method on bitter seeds to achieve a safe level of saponins while keeping the quality of the seeds.

## Data Availability Statement

The raw data supporting the conclusions of this article will be made available by the authors, without undue reservation.

## Author Contributions

KEH: conceptualization, methodology, formal analysis, software, writing—original draft, and writing-review and editing. MM: resources, project administration, and writing—review and editing. MLM: formal analysis, software, and writing—review and editing. KEK: project administration, funding acquisition, and validation. MS: writing—review and editing. MT: supervision, validation, and writing—review and editing. AY: conceptualization, supervision, validation, project administration, resources, and writing—review and editing. All authors contributed to the article and approved the submitted version.

## Conflict of Interest

The authors declare that the research was conducted in the absence of any commercial or financial relationships that could be construed as a potential conflict of interest.

## Publisher's Note

All claims expressed in this article are solely those of the authors and do not necessarily represent those of their affiliated organizations, or those of the publisher, the editors and the reviewers. Any product that may be evaluated in this article, or claim that may be made by its manufacturer, is not guaranteed or endorsed by the publisher.

## Abbreviations

WS: Washed seeds
NWS: Non-washed seeds
%ES: Percentage of eliminated saponins
SC: Saponins content
TPC: Total phenolic content
WHC: Water holding capacity
OHC: Oil holding capacity
SWC: Swelling capacity
EA: Emulsifying activity
BBD: Box–Benheken Design
RSM: Response surface methodology
X_1_: Water temperature
X_2_: Treatment time
X_3_: Volume of water.
